# JAK inhibitors: A new dawn for oral therapies in inflammatory bowel diseases

**DOI:** 10.3389/fmed.2023.1089099

**Published:** 2023-03-02

**Authors:** Claudia Herrera-deGuise, Xavier Serra-Ruiz, Ernesto Lastiri, Natalia Borruel

**Affiliations:** Unitat d’Atenció Crohn-Colitis, Digestive System Research Unit, Hospital Universitari Vall d’Hebrón, Barcelona, Spain

**Keywords:** inflammatory bowel diseases, JAK inhibitors, treatment, oral therapies, small molecules

## Abstract

Inflammatory bowel disease (IBD) is a chronic immune-mediated condition of the gastrointestinal tract that requires chronic treatment and strict surveillance. Development of new monoclonal antibodies targeting one or a few single cytokines, including anti-tumor necrosis factor agents, anti-IL 12/23 inhibitors, and anti-α4β7 integrin inhibitors, have dominated the pharmacological armamentarium in IBD in the last 20 years. Still, many patients experience incomplete or loss of response or develop serious adverse events and drug discontinuation. Janus kinase (JAK) is key to modulating the signal transduction pathway of several proinflammatory cytokines directly involved in gastrointestinal inflammation and, thus, probably IBD pathogenesis. Targeting the JAK-STAT pathway offers excellent potential for the treatment of IBD. The European Medical Agency has approved three JAK inhibitors for treating adults with moderate to severe Ulcerative Colitis when other treatments, including biological agents, have failed or no longer work or if the patient cannot take them. Although there are currently no approved JAK inhibitors for Crohn’s disease, upadacitinib and filgotinib have shown increased remission rates in these patients. Other JAK inhibitors, including gut-selective molecules, are currently being studied IBD. This review will discuss the JAK-STAT pathway, its implication in the pathogenesis of IBD, and the most recent evidence from clinical trials regarding the use of JAK inhibitors and their safety in IBD patients.

## 1. Introduction

Inflammatory bowel disease (IBD), which comprises Crohn’s disease (CD), and ulcerative colitis (UC), is characterized by chronic inflammation of the digestive tract. The etiology and pathogenesis of IBD remain unclear but likely implicate an interaction between environmental exposure, genetic risk, immunity, and gut microbiota (1). Cytokines play a crucial role in IBD pathogenesis by modulating the inflammatory cascade at numerous levels. An imbalance between pro-inflammatory and anti-inflammatory cytokines leads to disease perpetuation and tissue destruction by impeding the resolution of intestinal inflammation in IBD (2). Thus, research has long focused on identifying cytokines as potential targets for treating intestinal inflammation.

The introduction of biological therapies has revolutionized the management and outcomes of IBD. Infliximab, a tumor necrosis factor (TNF)-alpha receptor blocker, was the first monoclonal antibody approved for treating IBD in 1998. Three other anti-TNF molecules would follow adalimumab, golimumab, and certolizumab. Unfortunately, many patients show a poor response to these treatments. Immunogenicity through antibody production can also lead to the loss of response (3). Other pathways have been targeted, such as interleukin (IL) 12/23 axis and lymphocyte tracking using ustekinumab and vedolizumab, respectively. These agents are administered parenterally, which may burden healthcare systems and patients considerably (4). Therefore, there is a paramount need for drugs to maximize treatment efficacy while maintaining a good safety profile.

Janus kinase (JAK) inhibitors are a new therapeutic strategy in IBD. JAK inhibitors are the first IBD small molecule therapy and are administered orally. As they quickly enter the systemic circulation, they have a rapid onset of action and can induce fast clinical response (5). Compared to TNF-α inhibitors or anti-α4β7 integrin inhibitors that block a single or a few specific molecules, JAK inhibitors can block multiple cytokines from different inflammatory pathways simultaneously, thus potentially improving the therapeutic response (6). Dysregulation of JAK/STAT signaling has been described in numerous immune disorders, including IBD (7). JAK inhibitors do not elicit anti-drug antibodies; thus, immunogenicity is not an issue concerning JAK inhibitor therapy (8). This article aims to provide an overview of JAK inhibitors by presenting available information on already approved JAK inhibitors and those in clinical development for treating IBD.

## 2. JAK-STAT signaling pathway

**FIGURE 1:**
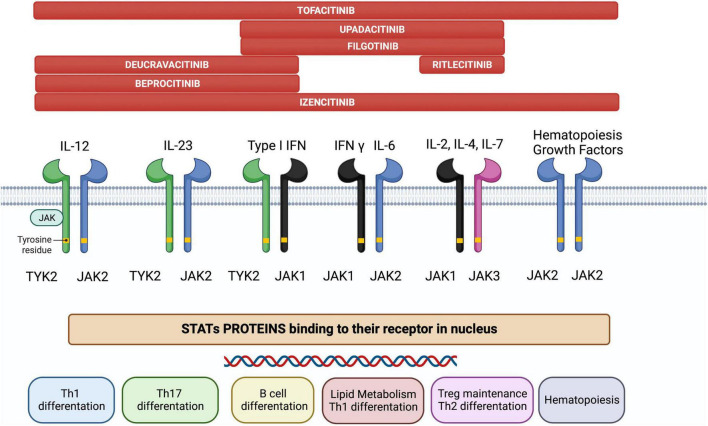
The JAK/STAT pathway constitutes a membrane-to-nucleus signaling module and induces the expression of various mediators of inflammation. Shown above are the different JAK inhibitors and their selectivity for different membrane JAK receptor subtypes; the immunological pathways inhibited by each receptor and their biological effects are shown below.

JAK is a family of intracellular tyrosine kinases, which include JAK1, JAK2, JAK3, and tyrosine kinase 2 (TYK2), that transduce cytokine-mediated signals *via* the STAT pathway (Figure 1). They are involved in multiple processes including immune and inflammatory responses (9).

Briefly, the JAK/STAT pathway works as follows (6): JAKs are activated by cytokine binding to its specific cell-surface receptor, which causes receptor dimerization and activation of their corresponding JAKs. JAKs then trigger phosphorylation in the cytoplasm and acts as a docking site for the STATs; docking of STATs causes their phosphorylation, dissociation from the receptor chains, dimerization with each other, and their eventual translocation to the cell nucleus. Gene transcription is activated, producing proteins implicated in immune response and inflammation pathways, creating and potentially aggravating a pro-inflammatory feedback loop.

JAK inhibitors suppress the JAK-STAT downstream signaling by interfering with the phosphorylation of JAK. Notably, specific JAK receptors show specificity for different cytokines. In IBD, cytokines associated with disease pathogenesis include IL-5, IL-9, IL-13, and IL-33 for UC, IL-10, IL-12, IL-27, and interferon (IFN)-γ for CD and IL-6, IL-12, IL-17, IL-21, IL-23, and TNF-α for both (10). Specifically, IL-23 activates JAK2 and the STAT3 pathway, and TYK2 activates the STAT4 pathway, whereas IL-6 activates JAK1, JAK2, and TYK2 *via* the STAT3 pathway (11). Since the inhibition of a specific JAK is related to a unique effect (12), the therapeutic possibilities of targeting the JAK-STAT pathway are vast.

## 3. JAK inhibitors

### 3.1. Tofacitinib

Although designed initially as a JAK3-specific inhibitor, tofacitinib is now considered a pan-JAK inhibitor since further studies demonstrated a binding affinity for JAK1 and, at higher doses, JAK2 (13).

Tofacitinib was the first JAK inhibitor approved by the FDA and EMA in 2018 for treating moderate-to-severe UC based on two induction trials and one 52-week maintenance trial (Table 1). The OCTAVE Induction 1 and 2 trials that included over 1,100 patients with moderate-to-severe UC unresponsive to conventional or anti-TNF treatment were randomized to receive oral tofacitinib 10 mg twice daily (bid) or placebo for 8 weeks (14). These studies showed that remission at week 8 was achieved by 18.5 and 16.6% of the patients receiving tofacitinib 10 mg bid vs. 8.2 and 3.6% of the patients with UC in the placebo group, respectively (*p* = 0.007 in OCTAVE Induction 1 and *p* < 0.001 in OCTAVE Induction 2) (Table 2). Furthermore, clinical response and mucosal healing rates at 8 weeks were higher in the tofacitinib arm. However, the incidence of infection (overall and severe) was significantly higher in the tofacitinib-treated group.

**TABLE 1:** JAK inhibitors approved or in current development for IBD.

Drug	Target	Gut selectivity	IBD type	Status
Tofacitinib	JAK1/JAK3	No	CD UC	Phase 2 completed (NCT00615199, NCT01393626) FDA/EMA approved
Peficitinib	JAK1/JAK3	No	CD UC	No studies Phase 2 completed (NCT01959282)
Upadacitinib	JAK1	No	CD UC	Phase 3 completed (NCT03345836), active not recruiting (NCT03345823) FDA/EMA approved
Filgotinib	JAK1	No	CD UC	Phase 3 active, not recruiting (NCT02914561), enrolling by invitation (NCT02914600) EMA approved, FDA rejected
Izencitinib (TD-1473)	pan-JAK	Yes	CD UC	Phase 2 terminated (NCT03635112) Phase 2b/3 terminated (NCT03758443)
Ivarmacitinib (SHR0302)	JAK1	No	CD UC	Phase 2 completed (NCT03677648) Phase 2 completed (NCT03675477)
OST-122 (Oncostellae)	JAK3/TYK2/ARK5	Yes	CD UC	No studies Phase 1b/2a recruiting (NCT04353791)
Deucravacitinib (BMS-986165)	TYK2	No	CD UC	Phase 2 recruiting (NCT03599622) Phase 2 active, not recruiting (NCT03934216)
Brepocitinib (PF-06700841)	JAK1/TYK2	No	CD UC	Phase 2 active, not recruiting (NCT03395184) Phase 2 completed (NCT02958865)
Ritlecitinib (PF-06651600)	JAK3	No	CD UC	Phase 2 active, not recruiting (NCT03395184) Phase 2 completed (NCT02958865)

**TABLE 2:** Major RCTs including JAK inhibitors for UC treatment.

Drug	RCT identifier	Patient number	Treatment phase	Duration	Dose	End point	Results
Tofacitinib	NCT01465763, NCT01458951 (14)	598 + 541	Induction	8 weeks	10 mg, bid	Clinical remission at week 8	In the OCTAVE Induction 1 trial, remission at 8 weeks occurred in 18.5% of the patients in the tofacitinib group versus 8.2% in the placebo group (*P* = 0.007); in the OCTAVE Induction 2 trial, remission occurred in 16.6% vs. 3.6 (*P* < 0.001).
	NCT01458574 (14)	593	Maintenance	52 weeks	5, 10 mg, bid	Clinical remission at week 52	In the OCTAVE Sustain trial, remission at 52 weeks occurred in 34.3% of the patients in the 5 mg tofacitinib group and 40.6% in the 10 mg tofacitinib group versus 11.1% in the placebo group (*P* < 0.001 for both comparisons with placebo).
	NCT03281304 (18)	140	Maintenance	6 months	5, 10 mg, bid	Clinical remission at month 6	Most patients in stable remission on 10 mg bid maintenance therapy maintained remission following dose de-escalation. For patients who dose de-escalated, those in deep endoscopic remission and those without prior TNFi failure were more likely to maintain remission.
Upadacitinib	NCT02819635, NCT03653026 (34)	474 + 522	Induction	8 weeks	45 mg, qd	Clinical remission at week 8	More patients achieved clinical remission with upadacitinib 45 mg (26% and 34 in UC1 and UC2, respectively) than in the placebo group (5% and 4 in UC1 and UC2, respectively; *p* < 0.0001).
	NCT02819635, NCT03653026 (34)	451	Maintenance	52 weeks	15, 30 mg, qd	Clinical remission at week 52	Clinical remission was achieved by more patients receiving upadacitinib 15 mg (42%) and upadacitinib 30 mg (52%) than those receiving placebo (12%; *p* < 0.0001).
Filgotinib	NCT02914522 (29)	2040	Induction	10 weeks	100, 200 mg, qd	Clinical remission at week 10	A greater proportion of patients given filgotinib 200 mg had clinical remission than those given placebo (induction study A 26.1 vs. 15.3%, *p* = 0.0157; induction study B 11.5% vs. 4.2, *p* = 0.0103). Clinical remission was not significantly different between filgotinib 100 mg and placebo at week 10.
	NCT02914522 (29)	664	Maintenance	58 weeks	100, 200 mg, qd	Clinical remission at week 58	37.2% of patients given filgotinib 200 mg had clinical remission versus 11.2% in the placebo group (*p* < 0.0001). Clinical remission was significantly different between filgotinib 100 mg and placebo by week 58 (23.8% vs. 13.5, *p* = 0.0420).

In a 2018 meta-analysis including biologic naive and non-naive UC patients, tofacitinib demonstrated the most relevant treatment effect in clinical remission (OR, 11.88; 95% CI, 2.32–60.89), and mucosal healing (OR, 4.7; 95% CI, 2.2–9.9) (15).

Patients with at least a partial clinical response to therapy after completing the OCTAVE Induction 1 or 2 trials were re-randomized to maintenance therapy with tofacitinib 10 or 5 mg bid or placebo for another 52 weeks in the OCTAVE Sustain trial. Remission was achieved in 34 and 41% of patients in the 5 mg and the 10 mg bid group, respectively, compared to 11% in the placebo group, with both dosages reaching statistical significance (*p* < 0.001). At week 52, patients receiving tofacitinib also achieved clinical response, sustained mucosal healing, and sustained steroid-free remission at higher rates than patients on placebo (14).

The OCTAVE Open (16) is a long-term extension, open-label trial evaluating the efficacy and safety of extended induction (16 weeks) with tofacitinib 10 mg bid in patients who did not respond to initial 8-week induction. Patients who achieved clinical response after 16 weeks were followed for another 36 months. The results showed that 52.2% of patients who did not achieve a clinical response by week 8 in the induction studies achieved a clinical response following extended induction. The rate of adverse effects was similar between both periods. At 12 months of OCTAVE Open, 70.3% of delayed responders maintained a clinical response, 56.8% achieved endoscopic improvement, and 44.6% endoscopic remission. At 36 months, 56.1, 52.0, and 44.6% maintained clinical response, endoscopic improvement, and remission, respectively.

Data from the meta-analysis by Taxonera et al. (17), including over 1,100 patients from 11 different studies, showed that at weeks 12–16, 47% of patients with UC reached clinical remission, 64.2% clinical response, 44.3% corticosteroid-free remission. And 48.3% mucosal healing at week 8. Those patients with no prior exposure to biologic agents showed a higher response rate at week 8 (OR 1.38; 95% CI 1.03–1.84). These results are concordant with those from clinical trials.

The RIVETING trial showed that most patients in stable remission on maintenance tofacitinib 10 mg bid could reduce the dose and remain in remission (18). Endoscopic remission and no previous failure to anti-TNF were associated with clinical remission during follow-up. Data was limited to the first 6 months.

Current guidelines advocate for tofacitinib in patients with moderately to severely active UC at an initial dose of 10 mg bid for at least 8 weeks, followed by a 5 mg bid of maintenance therapy. In patients not achieving remission after 8 weeks, prolonging the 10 mg bid dose can be considered for another 8 weeks. If a satisfactory therapeutic response after 16 weeks is not achieved, it should be considered a therapeutic failure, and tofacitinib discontinued (19).

For moderate-to-severe CD, tofacitinib 10 mg bid was studied in two phase II/IIB induction trials and one phase IIb maintenance trial (20). Tofacitinib failed to meet the primary endpoint of Crohn’s disease activity index of less than 150 points at 8 weeks from induction and maintenance at 26 weeks (Table 3).

**TABLE 3:** Major RCTs including JAK inhibitors for CD treatment.

Drug	RCT identifier	Patient number	Treatment phase	Duration	Dose	End point	Results
Tofacitinib	NCT00615199 (101)	139	Induction	4 weeks	1, 5, 15 mg, bid	Clinical response at week 4	A clinical response was observed in 36% (*p* = 0.467), 58% (*p* = 0.466), and 46% (*p* ≥ 0.999) of patients given the 1, 5, and 15 mg doses of tofacitinib, compared with 47% of patients given placebo.
	NCT01393626 (20)	280	Induction	8 weeks	5, 10 mg, bid	Clinical response-100 or clinical remission at week 8	At week 8 of induction, the proportion of patients with clinical remission was 43.5% and 43.0 with 5 and 10 mg twice daily, respectively, compared with 36.7% in the placebo group (*p* = 0.325 and 0.392 for 5 and 10 mg twice daily vs. placebo).
	NCT01393626 (20)	180	Maintenance	26 weeks	5, 10 mg, bid	Clinical response-100 or clinical remission at week 26	The proportion of patients with clinical response-100 or remission was 55.8% with tofacitinib 10 mg twice daily compared with 39.5% with tofacitinib 5 mg twice daily and 38.1% with placebo (*p* = 0.130 for 10 mg twice daily vs. placebo).
	NCT01470599 (102)	150	Maintenance	48 weeks	5 (patients in clinical remission), 10 mg (not in clinical remission), bid	Serious adverse effects and maintained remissions at week 48	Crohn’s disease worsening was the most frequent adverse event for tofacitinib 5 (33.9%) and 10 mg b.d. (19.3%). Patients not in remission at baseline, receiving 10 mg b.d., had higher rates of serious adverse events (19.3%) and discontinuation attributed to insufficient clinical response (30.7%) vs. 5 mg b.d. (8.1% and 9.7, respectively).
Upadacitinib	NCT02365649 (39)	220	Induction	16 weeks	3, 6, 12, 24 mg, bid; 24 mg, qd	Clinical remission at week 16 and endoscopic remission at week 12 or week 16	Upadacitinib did not significantly improve clinical remission at week 16 at any dose (with the exception of 6 mg at the *p* < 0.1 level). Endoscopic remission at week 12 was increased compared with placebo for doses of 3 mg (*p* < 0.1), 12 mg (*p* < 0.1), 24 mg bid (*p* < 0.01) and 24 mg qd (*p* < 0.05), in a dose-dependent manner.
	NCT02365649 (39)	180	Maintenance	52 weeks	3, 6, 12 mg, bid; 24 mg qd	Clinical and endoscopic remission at week 52	Patients receiving the 12 mg bid dose had the highest, although non-significant, responses compared with the other upadacitinib doses.
Filgotinib	NCT02048618 (30)	174	Induction	10 weeks	200 mg, qd	Clinical remission	60 (47%) of 128 patients treated with filgotinib 200 mg achieved clinical remission at week 10 vs. 10 (23%) of 44 patients treated with placebo (*p* = 0.0077).

#### 3.1.1. Acute severe ulcerative colitis

Acute severe ulcerative colitis (ASUC) is a life-threatening medical complication of UC. Patients with ASUC are hospitalized for rapid induction with intravenous corticosteroids. However, 30% of patients will not respond to corticosteroids alone and will require rescue treatment with ciclosporin or infliximab (21). Accelerated induction infliximab therapy has also decreased the need for colectomy in these patients. In a study evaluating an accelerated dosing strategy of infliximab, patients receiving three induction doses within 3 weeks had lower colectomy rates than standard dosing (6.7 vs. 40%; *p* = 0.039) (22). In another multicentre, retrospective study with over 200 ASUC patients, patients receiving a 10 mg/kg induction dose of infliximab presented a lower colectomy rate compared to the 5 mg/kg regime, although no difference was observed between regular and accelerated dosing strategies (23).

Promising results were first published regarding the use of tofacitinib as induction therapy for patients with ASUC; however, data was limited to small uncontrolled case series (24, 25). More recently, Berinstein et al. (26) performed a retrospective case-control study including over 150 biologic-experienced patients admitted with ASUC requiring intravenous corticosteroids. One-fourth of patients received tofacitinib and were matched to controls (*n* = 40 vs. *n* = 113). Patients received tofacitinib at either standard induction doses of 10 mg bid or an off-label dose of 10 mg three times daily (tid) for nine doses followed by 10 mg bid. The 10 mg tid posology was chosen based on the reported efficacy of 15 mg bid in the phase II trial and its short half-life of 3.2 h. Tofacitinib at an initial dose of 10 mg tid was protective against colectomy at 90 days compared with matched controls, whereas 10 mg bid was not. Rates of complications and steroid dependence were not different between groups. Larger prospective studies are much needed before making any solid recommendation regarding optimal dosing, duration, or safety of tofacitinib for ASUC treatment.

### 3.2. Filgotinib

Filgotinib (Jyseleca, Gilead, Galapagos) is an oral, once-daily JAK1 inhibitor (27). In late 2021, the EMA approved its use for the treatment of adult patients with moderate to severe UC when other treatments, including biological agents, have failed or no longer work or if the patient cannot take them. Filgotinib has an elimination half-life of 6 h, but it also produces an active metabolite with a half-life of up to 27 h (28). Both filgotinib and the active metabolite contribute to the clinical benefit of the molecule. Full pharmacodynamic effects are reached at a dose of 200 mg daily.

The phase IIb/III SELECTION trial assessed filgotinib efficacy for the treatment of UC, including two induction and one maintenance study (29). Patients were randomized to once-daily filgotinib at 200 or 100 mg dose or placebo. Patients in clinical remission or response in the induction studies at week 10 entered the maintenance study. Significantly more patients on filgotinib 200 mg were in clinical remission, defined as the composite of stool frequency, rectal bleeding, and endoscopic remission by weeks 10 and 58. At week 10, there was no difference in clinical remission between filgotinib 100 mg and placebo. However, by week 58, there was a significant difference in the filgotinib group. The adverse event rate was similar between the treatment and placebo groups. There were six cases of herpes zoster in the filgotinib group and none in the placebo group after 58 weeks of follow-up, four in the filgotinib 200 mg and two in the 100 mg group.

The phase II FITZROY study examined the efficacy and safety of filgotinib for the treatment of moderate-to-severe CD (30). Enrolled patients were randomized to receive filgotinib 200 mg or a placebo for 10 weeks. According to their response, patients were re-assigned to filgotinib 200 mg, filgotinib 100 mg, or placebo for an additional 10 weeks. More patients that received filgotinib achieved clinical remission (*p* = 0.0077; 47 vs. 23%). The clinical remission rate at week 10 was higher among anti-TNF naïve patients. There was a numerically higher difference in all filgotinib-treated groups in both endoscopic response and mucosal healing. Differences versus placebo did not reach statistical significance beyond this period. Serious infections were reported in 3% of filgotinib-treated patients and none in the placebo group.

A phase III study to evaluate the efficacy and safety of filgotinib in CD is currently ongoing [NCT02914561 (31), NCT02914600 (32)], as is a phase II trial assessing filgotinib in perianal fistulizing CD (NCT03077412) (33).

### 3.3. Upadacitinib

The FDA and the EMA have recently approved Upadacitinib (Rinvoq, AbbVie) for treating adult patients with moderate to severe UC when other treatments, including biological agents, have failed or no longer work or if the patient cannot take them. Upadacitinib shows increased selectivity for JAK1 compared with JAK2, JAK3, and TYK2. It is metabolized mainly in the liver, eliminated by renal excretion, and has a half-life of 4 h. Data from U-ACHIEVE induction (UC1) and U-ACCOMPLISH (UC2), and one maintenance study, U-ACHIEVE maintenance (UC3), supported its approval. These phase III trials randomly assigned patients with moderate to severe UC to upadacitinib 45 mg daily or placebo for 8 weeks (induction studies) (34). Responders were re-randomized to maintenance doses of 15 and 30 mg or placebo for an additional 52-week period (maintenance study).

Results of the UC1 and UC2 induction studies showed that at week 8, remission was reached by 26 and 34% of patients receiving upadacitinib 45 mg vs. 5 and 4% of patients in the placebo group [21.6% (adjusted treatment difference); 15.8–27.4 for UC1 and 29%; 23.2–34.7 for UC2].

Interestingly, the definition of clinical remission used by the authors is quite more stringent than in previous studies as only a rectal bleeding score of 0 is considered remission, while Physician’s Global Assessment (PGA) is excluded from Mayo score due to its subjectiveness. Also, the criteria for mucosal healing requiring both endoscopic and histological remission, defined as endoscopic score of 0 and a Geboes score < 2, were also more stringent compared to previous studies. Upadacitinib 45 mg has shown to improve UC symptoms as early as day 1, thus providing patients with fast symptom relief (35). Furthermore, early symptom improvement has been associated with clinical remission or clinical response by week 8. Moreover, the TOUR study showed significant and persistent improvement in UC disease activity patient reported outcomes (PROs) as soon as day 3 in a real-world setting (36).

In the maintenance study, more patients taking upadacitinib achieved remission than those on placebo (30.7% for upadacitinib 15 mg and 39% for upadacitinib 30 mg vs. 12% placebo). Safety results in UC were consistent with the safety profile of upadacitinib, with no new significant safety risks reported. The most commonly reported adverse reactions were nasopharyngitis, creatine phosphokinase elevation, and acne. Thrombotic events, major cardiovascular events, malignancy excluding non-melanoma skin cancer, and gastrointestinal perforation were infrequently reported.

Recently, two systematic review and network meta-analyses have been published with upadacitinib ranking highest for the induction of clinical remission in UC (37, 38). In Burr’s meta-analysis, upadacitinib also ranked first for endoscopic improvement in patients who had received anti-TNF before. Interestingly, upadacitinib ranked highest for adverse effects in both studies.

The phase II CELEST trial evaluated the efficacy and safety of upadacitinib in CD patients. This study assessed multiple doses of upadacitinib as an induction treatment until week 16, followed by blinded extension therapy for 36 weeks (39). Clinical remission at week 16 and endoscopic remission at 12 or 16 weeks were assessed. Upadacitinib did not improve clinical remission rates at week 16 at any tested dose (except for the 6 mg dose at the *p* < 0.1 level). However, upadacitinib did improve endoscopy remission at week 12 compared with placebo for the 3 mg, 12 mg, 24 mg bid, and 24 mg qd (once daily) doses in a dose-dependent manner. Efficacy was maintained for most study endpoints through week 52. Regarding the safety profile of upadacitinib, more infections and severe infections were observed compared to placebo, including three cases of herpes zoster.

Results from the phase III U-EXCEED and U-EXCEL induction studies and the U-ENDURE maintenance study are awaited [NCT03345836 (40), NCT03345823 (41)].

In a recent systematic review and network meta-analysis, upadacitinib 45 mg ranked third in the induction of clinical remission in CD patients. Moreover, upadacitinib 30 mg once daily ranked first in terms of maintenance of clinical remission (42).

### 3.4. Peficitinib

Peficitinib (ASP015K, Astellas Pharma) is a JAK1/JAK3 inhibitor that is metabolized in the liver and whose half-life is 2.8–13 h (28). Safety and efficacy of peficitinib were evaluated in a phase IIb trial where patients with moderate-to-severe UC were randomized to receive peficitinib 25, 75, or 150 mg once daily, or peficitinib 75 mg twice daily, or placebo (43). The primary endpoint established as a dose-response to peficitinib at week 8 was not achieved. No more trials regarding peficitinib are currently in progress.

### 3.5. Izencitinib

Izencitinib (TD-1473, Theravance Biopharma) is an oral gut-selective pan-JAK inhibitor intended to lower the systemic toxicity of pan-JAK inhibition. Sandborn et al. (44) demonstrated in a phase 1b study that oral TD-1473 administration achieved high, biologically active colonic tissue exposure with low plasma concentrations. Interestingly, TD-1473 showed approximately 40-fold stronger potency for TYK2 compared to tofacitinib. IL-12 and IL-23 signal *via* JAK2 and TYK2, and to a much lesser extent for JAK1 or JAK3. A molecule with higher avidity for TYK2 (or JAK2) could prove more effective, especially in CD, as seen for ustekinumab, by inhibiting IL-12 and IL-23 (45).

For UC, an 8-week phase IIb dose-finding induction study failed to reach the primary endpoint defined as a change in the total Mayo score at week 8 relative to placebo (NCT03758443) (46). The safety and efficacy data of this phase IIb study were planned to inform induction and maintenance dose regimens for a confirmatory phase III induction study and the ongoing maintenance study. A phase II study of izencitinib in moderately to severely active CD was recently finished, pending results (NCT03635112) (47).

### 3.6. Ivarmacitinib

Ivarmacitinib (formerly SHR0302) (Reistone Biopharma Company Limited) is a selective JAK1 inhibitor. Ivarmacitinib has been shown to be at least 10 times more selective for JAK1 than for JAK2, and 77 and 420 times more selective for JAK1 than for JAK3 and TYK2, respectively (48).

The AMBER2 phase II studied Ivarmacitinib’s safety profile and efficacy (49). Doses of 8 mg once daily (OD), 4 mg bid, and 4 mg OD in moderate-to-severe UC patients were explored. Patients received ivarmacitinib for 8 weeks in the induction phase and remained in the same treatment group for a further 8 weeks (extension phase); patients receiving placebo were randomized to one of the three active treatment groups. The primary endpoint was the clinical response rate at week 8, and it was significantly higher in the 8 mg OD (46.3%), 4 mg bid (46.3%), and 4 mg OD (43.9%) groups than in placebo (26.8%). Clinical remission rates were also significantly higher for all ivarmacitinib groups. There is an ongoing phase 3 study with Ivarmacitinib for UC (NCT05181137) (50), for which patients are still being recruited.

Recently, a phase II study to investigate ivarmacitinib in patients with Crohn’s Disease (NCT03677648) (51) has been completed; this is a 12 + 12 weeks study in which patients complete the first 12-week treatment phase and enter a blinded arm in the 12-week extension phase. Results are pending.

### 3.7. OST-122

OST-122 (Oncostellae) is an oral, gut-restricted, and subtype-selective Jak3/Tyk2/Ark5 inhibitor designed for the local treatment of UC, CD, and fibrotic lesions in CD. Tolerability of the drug was adequate in a phase 1 study in healthy volunteers and proved stability during the gastrointestinal transit, with no significant plasma levels detected. The gut-restricted pharmacokinetic profile of OST-122 is theorized to lower the risk of systemic toxicities compared to other JAK inhibitors. NCT04353791 (52) is a phase Ib/IIa study to evaluate the safety and tolerability of treatment with OST-122 in patients with moderate-to-severe UC over 28 days. There is no available data from this study.

### 3.8. Deucravacitinib

Deucravacitinib (BMS-986165, Bristol-Myers Squibb) inhibits TYK2, which modulates IL-12 and IL-23 pathways, by binding selectively to the JH2 pseudokinase domain and not to the active catalytic site of TYK2 (53). Deucravacitinib effectively treated psoriasis in a phase II trial (54). Two phase II, randomized, double-blinded, placebo-controlled clinical trials to investigate the efficacy and safety of deucravacitinib in patients with moderate-to-severe CD (NCT03599622; LATTICE study) (55) and UC (NCT03934216; LATTICE-UC) (56) are currently in progress.

### 3.9. Other JAK inhibitors

Brepocitinib (PF-06700841) (Pfizer) is a selective TYK2 (IL-12 and IL-23) and JAK1 inhibitor designed to improve outcomes in clinical efficacy and safety compared to other JAK inhibitors (e.g., erythropoietin modulation) (57).

Ritlecitinib (PF-06651600) (Pfizer) is a selective JAK3 inhibitor designed to be highly effective in γc-cytokine signaling inhibition while preserving the JAK1-dependent anti-inflammatory signaling (58).

NCT03395184 (59) and NCT02958865 (60) are two phase II randomized, double-blind, placebo-controlled, parallel-group studies of PF-06651600 and PF-06700841 as induction and maintenance therapy in patients with moderate-to-severe CD and UC, respectively. NCT02958865 has been completed, pending results. NCT03395184 is stated as active but has yet to recruit patients.

## 4. Safety and adverse effects

### 4.1. Infections

JAK inhibitors are associated with a high risk of infection by the herpes zoster virus (HZ). A pooled *post hoc* analysis from induction, maintenance, and open-label studies in UC patients treated with tofacitinib showed that 5.6% developed HZ. The incidence rate (IR) was 4.07 over a mean of nearly 2 years. The risk factors were patients 65 years or older, 9.55; Asian race, 6.49; prior anti-TNF failure; and tofacitinib 10 mg bid dose, 4.25. Older age and previous anti-TNF failure were the only independent risk factors in the multivariate analysis (61).

The primary immune response to HZ virus comes from the type I and II IFN pathway, facilitated and transmitted at the transmembrane level by various JAK pathways such as JAK1-TYK2 and JAK1-JAK2. Their signaling activates the STAT proteins system (62). In a recent network meta-analysis (63), tofacitinib, and others JAK inhibitors increase the risk of herpes zoster infection even with low doses. The higher risk was specifically with tofacitinib 10 mg bid (RR = 6.90; 95% CI 1.56–30.63) and upadacitinib 45 mg o.d. (RR = 7.89; 95% CI 1.04–59.59).

In phase III clinical trials for filgotinib in UC, HZ was reported in one case in the 200 mg group and one in the 100 mg group (64). In a pooled analysis of patients with RA, the 200 mg treated patients had more risk than the 100 mg group, with an IR of 8.7. A previous history of HZ, Asian race, and age ≥ 50 years were associated with increased risk (65).

Three HZ cases were reported during the induction period of upadacitinib in CD patients and one in the upadacitinib UC trial (39).

Thus, it is recommended that the adjuvanted recombinant HZ subunit vaccine (Shingrix) be administered intramuscularly in two doses 2 months apart to prevent HZ in patients older than 50. Live vaccines (Zostavax^®^) are contraindicated in patients under immunosuppressive therapy, including JAK inhibitors (66, 67).

### 4.2. Hyperlipidaemia

Tofacitinib can cause a reversible rise in serum levels of lipids, mainly in the first 6 weeks. After 4–8 weeks of treatment, the levels remain stable and can return to baseline upon cessation of the drug (68). Previous studies on IBD have shown that inflammation can lower lipid levels; therefore, controlling the inflammatory response may result in higher levels (69).

A phase I study in RA patients receiving tofacitinib showed that the cholesterol ester fractional catabolic rate was higher in RA compared to healthy subjects. The cholesterol ester production and cholesterol efflux rate were similar (70). In a recent real-world evidence study in Spain, the incidence of hypercholesterolemia was 6.6% from the total cohort (71).

A *post hoc* analysis of 22 RCTs assessing MACEs (major adverse cardiovascular events) JAK inhibitor-treated patients showed a RR of MACEs of 1.07 (95% CI, 0.56–2.03). When only placebo-controlled RCTs were analyzed, a RR of 1.09 (95% CI, 0.54–2.21) was observed (72). There was a similar incidence of MACEs in the UC tofacitinib studies compared to RA studies (MACE IR, 0.37 in OLE studies) (73).

Data from the pivotal clinical trials demonstrated that at 8 weeks, there were more critical rises in total cholesterol (TC), high-density lipoprotein cholesterol (HDL-C), and low-density lipoprotein cholesterol (LDL-C) in tofacitinib-treated patients. C-reactive protein was inversely correlated with lipid levels. The Reynolds Risk Score estimates the 10-year cardiovascular events, taking into account the C-reactive protein and other traditional risk factors was the same in patients treated with tofacitinib or placebo (74).

Data from studies of patients with RA receiving upadacitinib moderate increases in lipid levels were associated with a significantly better response to clinical activity; there are no studies regarding cardiovascular risk assessment as tofacitinib (75). In the pivotal studies for upadacitinib, it was observed that total cholesterol concentrations were increased in all treatment arms. In contrast, the ratio of low-density lipoprotein and high-density lipoprotein cholesterol remained normal (34). In filgotinib studies, during induction, there was a modest increase in total fasting cholesterol, LDL, and HDL in all treatment groups. However, during maintenance, there was no elevation of blood lipids in the filgotinib groups, and the levels remained stable (29).

In a study with PsA treated with filgotinib, TC, LDL-C, and HDL-C levels increased versus baseline, resulting in a decreased TC/HDL-C ratio (76).

Patients should have a baseline fasting lipid profile and another determination 4–8 weeks after starting treatment and every 6 months after that. If additional CV risk factors are detected, lipid-lowering agents should be started (67, 77).

### 4.3. Venous thromboembolism

FDA and EMA recommend avoiding JAK inhibitors in patients at risk for venous thromboembolism (VTE), including deep vein thrombosis (DVT) and PE (pulmonary embolism). The ORAL Surveillance safety study of tofacitinib in patients with RA ≥ 50 years old, with one or more cardiovascular risk factors, showed a higher risk of PE in patients on tofacitinib 10 mg bid compared to patients with an anti-TNF (78).

There are confounding factors in calculating the actual risk of JAK inhibitors in VTE. The risk factors that raise the risk of DVT, PE, and ATE are; a previous history of VTE, a hypercoagulable state, reduced mobility, and recent major surgery or trauma. Smoking, MI in the previous 3 months, age higher than 50 years, malignancy, obesity, use of combined oral contraceptives or hormonal replacement therapy, and long flights have also been described (66, 67). However, patients with chronic inflammatory diseases, like untreated RA or IBD patients with active disease, may also have a higher risk for venous thrombosis.

The safety and efficacy of tofacitinib were described in the OCTAVE Open study (79). The authors concluded that tofacitinib had an acceptable safety profile during long-term therapy for UC. The IRs for thromboembolic events in tofacitinib-treated patients correspond to those reported for UC patients in general. However, the label for tofacitinib in UC was updated to include this risk.

In a *post hoc* study of patients with UC tofacitinib-treated, there was a report of one VTE case and four PE events; during the study, all VTE cases were in patients on a 10 mg bid dose (61). It is recommended that the use of a 10 mg bid of tofacitinib for more than 3 months should be avoided, especially in high-risk VTE patients, to decrease that risk (66). However, long-term safety data on UC patients on the 10 mg bid dose is needed.

The incidence rate of VTE for upadacitinib was 1.1 per 100 patient-years; in the case of filgotinib, one PE episode was reported in the 100 mg dose arm, and two DVTs were reported in the placebo arm (80, 81). At this moment, it is unknown if this is a drug class adverse event or due to inhibition of a specific pathway, data that must be confirmed in long-term studies; therefore, it cannot be established that selective JAK-1 inhibition is a measure to decrease the risk of VTE.

### 4.4. Cytopenias

Tofacitinib can induce a mild and transient decrease in blood cell counts, which are normalized over time in long-term follow-up studies. In a trial in patients with RA, IR for neutropenia and lymphopenia were 0.52 and 1.11; however, no patients developed severe infections within the month of their lowest neutrophil count. In patients with severe lymphopenia (defined as < 0.5 × 103 cells/mm^3^), 5 cases had associated severe infections (82). The theoretical mechanism is the JAK2 signaling blockade that intervenes in hematopoiesis. Izencitinib, also a pan-JAK inhibitor, could potentially cause pancytopenia, although there are no reports.

## 5. Pregnancy and breastfeeding

Due to the scarce data on pregnant patients with IBD, current European Crohn’s and Colitis Organisation (ECCO) guidelines recommend that JAK inhibitors are contraindicated (83). Contraception during treatment with tofacitinib and 4–6 weeks after the last dose is recommended by the manufacturer (84). Tofacitinib is assumed to cross the placenta as a small molecule. There is a report of 11 cases of maternal and 14 cases of paternal exposure to tofacitinib before or during pregnancy; the outcomes were 15 healthy newborns, 2 spontaneous abortions, 2 medical terminations but no congenital malformation or fetal/newborn fatalities (85).

There are reports in filgotinib-treated rats where a decreased male fertility was observed due to impaired spermatogenesis, but there was no effect on female fertility (86). The MANTA and MANTA-RAy are two studies with men with active IBD and rheumatic diseases, respectively, that are ongoing to evaluate the proportion of patients with ≥ 50% decrease from baseline in sperm concentration at week 13, comparing filgotinib 200 mg vs. placebo; they informed in an interim report that there are not significant differences in sperm concentration between both groups (filgotinib 6.7%, placebo 8.3%) (87).

Because there is currently no solid evidence of its safety, JAK inhibitors should be avoided in women seeking pregnancy. Furthermore, tofacitinib should be discontinued for at least 1 week before conception, given its short half-life. Regarding breastfeeding, the current recommendation is not to breastfeed if possible or to wait for a least 18 h after tofacitinib intake (83).

## 6. Discussion

Targeting the JAK-STAT pathway offers great potential for IBD. Tofacitinib, upadacitinib, and filgotinib are approved treatments for moderately to severely active UC. UC patients who had not responded to conventional therapy or biologics were treated with tofacitinib and had a higher rate of clinical remission, clinical response, and mucosal healing at week 8 in phase III studies. Furthermore, UC patients who received tofacitinib as maintenance therapy had a higher remission rate at week 54. By contrast, clinical trials of tofacitinib for CD have been disappointing, as no differences in response or remission at various doses have been shown compared with placebo. However, the selective JAK1 inhibitors upadacitinib and filgotinib showed increased remission rates in patients with moderate to severe CD. Other JAK inhibitors, including gut-selective molecules, are complete or ongoing for UC and CD. JAK inhibitors have undoubtedly revolutionized the IBD landscape as they provide clinicians with agents characterized by their rapid mechanism of action and oral administration, generally well received by patients. However, current drug development requires not only effectiveness but a reasonable safety profile at the same time. Accordingly, there is a growing trend toward personalized therapy, where the specific patient’s needs are met with tailored treatment according to the patient’s characteristics and preferences.

One of the biggest concerns regarding JAK inhibitors is cardiovascular and VTE risks. To date, most safety data come from RA studies, in which MACE and cancer rates were increased with the use of tofacitinib compared to anti-TNF agents. How valid the extrapolation of safety data from RA to UC has often been questioned based on the different etiopathogenesis and patients’ characteristics of these two entities, such as an older age of disease onset and a higher rate of comorbidities in the case of RA. Moreover, no increased risk of VTE, PE, and DVT events was seen in patients on JAK inhibitors with immune-mediated inflammatory diseases in a recent meta-analysis (88).

Current guidelines and recent EMA advice (89) advocate for minimizing the potential serious side effects of JAK inhibitors and restricting their use in those aged 65 years or above, those at increased risk of major cardiovascular conditions and cancer, and those who smoke or have done so for a long time. It makes sense that in the case of using any JAK inhibitors in this population, the lowest possible dose should be used to minimize potential side effects of these drugs. The warning applies to all licensed JAK inhibitors in Europe regardless of their indication (tofacitinib, abrocitinib, baricitinib, upadacitinib, filgotinib).

Of special mention is the higher risk of herpes zoster among patients treated with JAK inhibitors. Interestingly, a meta-analysis showed a higher relative risk of herpes zoster in those patients receiving tofacitinib compared to filgotinib or upadacitinib (72). Whether JAK-1’s selectivity of upadacitinib and filgotinib is relevant in terms of overall security, compared to other pan-JAK inhibitor options, remains to be seen.

The global tofacitinib UC clinical program analysis, including data with up to 7.8 years of tofacitinib exposure, showed consistency with the previously known safety profile of tofacitinib in UC (90). Unfortunately, dose dependency could not be thoroughly evaluated as most patients were on the 10 mg bid dose.

Fast onset of action is critical in UC patients with a severe disease where corticosteroid-sparing is a priority. The TOUR study shows real-world data on the speed of action of tofacitinib in UC. Administration route preference should be considered as long as JAK inhibitors are a valid therapeutic option for a specific patient. The patient’s medical record is relevant, not only concerning those factors that might guide clinicians toward another therapeutic alternative (elderly age, previous thromboembolic or cancer disease, smoking status, as exposed above), but also the presence of rheumatological manifestations of IBD, where JAK inhibitors are an excellent option to cover both entities.

Tofacitinib is currently approved in Europe for treating RA, psoriatic arthritis, ankylosing spondylitis, and polyarticular juvenile idiopathic arthritis, as well as UC. However, one should consider that positive results in treating other immune-mediated inflammatory diseases cannot be directly applied to extraintestinal manifestations management. In a *post hoc* analysis of the OCTAVE Induction 1 and 2 and OCTAVE Sustain, only the tofacitinib-treated patients showed improvement in peripheral arthritis at week 52 (91). Phase III studies showed the effectiveness of tofacitinib over placebo in ankylosing spondylitis, suggesting it may also be efficacious in axial spondyloarthropathy (92), unlike ustekinumab which has proven ineffective (93). Tofacitinib has also been successfully used to treat refractory uveitis, scleritis (94), and pyoderma gangrenosum (95). These findings situate tofacitinib and, by extension, JAK inhibitors as the preferred second-line therapy in patients with EIMs.

There is already growing evidence regarding real-world data on all the approved JAK. In a systematic review and meta-analysis comprising 830 patients and nine studies (93), tofacitinib achieved induction of clinical response and remission rates at week 8 of 51 and 37%, respectively, and maintenance of clinical response and remission rates at week 24 of 40 and 29%, respectively. There are already a few small cohorts of real-world patients using upadacitinib for both UC (94) and CD (95) with promising results.

Recently, Chugh et al. published their real-world experience with upadacitinib in CD. In total, 36 of all 45 CD patients included in the analysis received the agent for the CD indication (96). Upadacitinib was effective in inducing remission. No new safety issues were observed.

How to position JAK inhibitors into the IBD therapeutic algorithms remains a matter of debate. To date, anti-TNF molecules are the most commonly used first-line agents. Considering the high anti-TNF therapy failure rates, potential contraindications, and adverse effects, the expansion of the therapeutic armamentarium for IBD is paramount.

In a recent real-world study including patients with anti-TNF and vedolizumab failure, tofacitinib and ustekinumab were equally effective as third-line biologics in inducing steroid-free clinical remission at 12–16 weeks (97).

In another retrospective study comparing the effectiveness of tofacitinib and ustekinumab after anti-TNF failure, no differences were observed in steroid-free clinical remission at either week 52, and drug survival rates were similar (98).

Finally, data analysis from the nationwide Dutch Registry cohort showed superior effectiveness of tofacitinib compared to vedolizumab in anti-TNF experienced UC patients, along with comparable safety outcomes (99).

Further studies are warranted to guide the positioning of biologics and JAK inhibitors in patients with anti-TNF refractory UC.

There is no data on the use of JAK inhibitors in patients failing a previous JAK inhibitor in IBD. Large prospective studies are needed to demonstrate the superiority of one JAK inhibitor over other, as inferred from network meta-analyses. Such an approach may be reasonable when a partial clinical and/or endoscopic response has been reached without achieving the final therapeutic goal. There is emerging evidence from RA patients. In a real-world population of RA, including four hundred treatment courses of JAK inhibitors, switching to another JAK inhibitor resulted in higher drug retention than switching to an anti-TNF (100). In this study, tofacitinib was the most commonly used initial JAK inhibitor (83.2%), whereas baricitinib and upadacitinib were the most common second-line options.

JAK inhibitors are a relatively new therapeutic strategy to treat UC and CD, characterized by an oral route of administration, short half-life, and fast onset of action. Large, prospective head-to-head trials, including JAK inhibitors and biologics, are eagerly awaited to position these molecules into the current therapeutic algorithm.

## Author contributions

CH-d and NB: conceptualization, design, and review and editing. XS-R and EL: data collection and writing and editing. All authors contributed to the article and approved the submitted version.

## References

[1] Goethel A Croitoru K Philpott D. The interplay between microbes and the immune response in inflammatory bowel disease. *J Physiol.* (2018) 596:3869–82. 10.1113/JP275396 29806140 PMC6117586

[2] Neurath M. Cytokines in inflammatory bowel disease. *Nat Rev Immunol.* (2014) 14:329–42. 10.1038/nri3661 24751956

[3] Moss A Brinks V Carpenter J. Review article: immunogenicity of anti-tnf biologics in ibd - the role of patient, product and prescriber factors. *Aliment Pharmacol Ther* (2013) 38:1188–97. 10.1111/apt.12507 24118102

[4] Harris C Cummings J. Jak1 inhibition and inflammatory bowel disease. *Rheumatology (Oxford).* (2021) 60(Supple 2):ii45–51. 10.1093/rheumatology/keaa896 33950226 PMC8098109

[5] Tanaka Y Luo Y O’Shea J Nakayamada S. Janus kinase-targeting therapies in rheumatology: a mechanisms-based approach. *Nat Rev Rheumatol.* (2022) 18:133–45. 10.1038/s41584-021-00726-8 34987201 PMC8730299

[6] Fernandez-Clotet A Castro-Poceiro J Panes J. Jak inhibition: the most promising agents in the IBD pipeline? *Curr Pharm Des.* (2019) 25:32–40. 10.2174/1381612825666190405141410 30950344

[7] Shuai K Liu B. Regulation of jak-stat signalling in the immune system. *Nat Rev Immunol.* (2003) 3:900–11. 10.1038/nri1226 14668806

[8] Shivaji U Nardone O Cannatelli R Smith S Ghosh S Iacucci M. Small molecule oral targeted therapies in ulcerative colitis. *Lancet Gastroenterol Hepatol.* (2020) 5:850–61. 10.1016/S2468-1253(19)30414-5 32171056

[9] Ghoreschi K Laurence A O’Shea J. Janus kinases in immune cell signaling. *Immunol Rev.* (2009) 228:273–87. 10.1111/j.1600-065X.2008.00754.x 19290934 PMC2782696

[10] Nemeth Z Bogdanovski D Barratt-Stopper P Paglinco S Antonioli L Rolandelli R. Crohn’s disease and ulcerative colitis show unique cytokine profiles. *Cureus.* (2017) 9:e1177. 10.7759/cureus.1177 28533995 PMC5438231

[11] Salas A Hernandez-Rocha C Duijvestein M Faubion W McGovern D Vermeire S Jak-Stat pathway targeting for the treatment of inflammatory bowel disease. *Nat Rev Gastroenterol Hepatol.* (2020) 17:323–37. 10.1038/s41575-020-0273-0 32203403

[12] O’Sullivan L Liongue C Lewis R Stephenson S Ward A. Cytokine receptor signaling through the jak-stat-socs pathway in disease. *Mol Immunol.* (2007) 44:2497–506. 10.1016/j.molimm.2006.11.025 17208301

[13] Ghoreschi K Jesson M Li X Lee J Ghosh S Alsup J Modulation of innate and adaptive immune responses by tofacitinib (Cp-690,550). *J Immunol.* (2011) 186:4234–43. 10.4049/jimmunol.1003668 21383241 PMC3108067

[14] Sandborn W Su C Sands B D’Haens G Vermeire S Schreiber S Tofacitinib as induction and maintenance therapy for ulcerative colitis. *N Engl J Med.* (2017) 376:1723–36. 10.1056/NEJMoa1606910 28467869

[15] Singh S Fumery M Sandborn W Murad M. Systematic review with network meta-analysis: first- and second-line pharmacotherapy for moderate-severe ulcerative colitis. *Aliment Pharmacol Ther.* (2018) 47:162–75. 10.1111/apt.14422 29205406

[16] Sandborn W Peyrin-Biroulet L Quirk D Wang W Nduaka C Mukherjee A Efficacy and safety of extended induction with tofacitinib for the treatment of ulcerative colitis. *Clin Gastroenterol Hepatol.* (2022) 20:1821–30.e3. 10.1016/j.cgh.2020.10.038 33127596

[17] Taxonera C Olivares D Alba C. Real-World effectiveness and safety of tofacitinib in patients with ulcerative colitis: systematic review with meta-analysis. *Inflamm Bowel Dis.* (2022) 28:32–40. 10.1093/ibd/izab011 33586766

[18] Vermeire S Su C Lawendy N Kobayashi T Sandborn W Rubin D Outcomes of tofacitinib dose reduction in patients with ulcerative colitis in stable remission from the randomised riveting trial. *J Crohns Colitis.* (2021) 15:1130–41. 10.1093/ecco-jcc/jjaa249 33290538 PMC8256630

[19] Feuerstein J Isaacs K Schneider Y Siddique S Falck-Ytter Y Singh S Aga clinical practice guidelines on the management of moderate to severe ulcerative colitis. *Gastroenterology.* (2020) 158:1450–61. 10.1053/j.gastro.2020.01.006 31945371 PMC7175923

[20] Panes J Sandborn W Schreiber S Sands B Vermeire S D’Haens G Tofacitinib for induction and maintenance therapy of Crohn’s disease: results of two phase iib randomised placebo-controlled trials. *Gut.* (2017) 66:1049–59. 10.1136/gutjnl-2016-312735 28209624 PMC5532457

[21] Turner D Walsh C Steinhart A Griffiths A. Response to corticosteroids in severe ulcerative colitis: a systematic review of the literature and a meta-regression. *Clin Gastroenterol Hepatol.* (2007) 5:103–10. 10.1016/j.cgh.2006.09.033 17142106

[22] Gibson D Heetun Z Redmond C Nanda K Keegan D Byrne K An accelerated infliximab induction regimen reduces the need for early colectomy in patients with acute severe ulcerative colitis. *Clin Gastroenterol Hepatol.* (2015) 13:330–5.e1. 10.1016/j.cgh.2014.07.041 25086187

[23] Nalagatla N Falloon K Tran G Borren N Avalos D Luther J Effect of accelerated infliximab induction on short- and long-term outcomes of acute severe ulcerative colitis: a retrospective multicenter study and meta-analysis. *Clin Gastroenterol Hepatol.* (2019) 17:502–9.e1. 10.1016/j.cgh.2018.06.031 29944926 PMC6309670

[24] Kotwani P Terdiman J Lewin S. Tofacitinib for rescue therapy in acute severe ulcerative colitis: a real-world experience. *J Crohns Colitis.* (2020) 14:1026–8. 10.1093/ecco-jcc/jjaa018 32020189

[25] Berinstein J Steiner C Regal R Allen J Kinnucan J Stidham R Efficacy of induction therapy with high-intensity tofacitinib in 4 patients with acute severe ulcerative colitis. *Clin Gastroenterol Hepatol.* (2019) 17:988–90.e1. 30458248 10.1016/j.cgh.2018.11.022 PMC7194692

[26] Berinstein J Sheehan J Dias M Berinstein E Steiner C Johnson L Tofacitinib for biologic-experienced hospitalized patients with acute severe ulcerative colitis: a retrospective case-control study. *Clin Gastroenterol Hepatol.* (2021) 19:2112–20.e1. 10.1016/j.cgh.2018.11.022 34048936 PMC8760630

[27] Dowty M Lin T Jesson M Hegen M Martin D Katkade V Janus kinase inhibitors for the treatment of rheumatoid arthritis demonstrate similar profiles of in vitro cytokine receptor inhibition. *Pharmacol Res Perspect.* (2019) 7:e00537. 10.1002/prp2.537 31832202 PMC6857076

[28] Garrido I Lopes S Macedo G. Hit the road Jak! the role of new oral treatment in inflammatory bowel disease. *Inflamm Bowel Dis.* (2021) 27:2010–22. 10.1093/ibd/izab037 33742651

[29] Feagan B Danese S Loftus E Jr. Vermeire S Schreiber S Ritter T Filgotinib as induction and maintenance therapy for ulcerative colitis (selection): a phase 2b/3 double-blind, randomised, placebo-controlled trial. *Lancet.* (2021) 397:2372–84. 10.1016/S0140-6736(21)00666-8 34090625

[30] Vermeire S Schreiber S Petryka R Kuehbacher T Hebuterne X Roblin X Clinical remission in patients with moderate-to-severe Crohn’s disease treated with filgotinib (the fitzroy study): results from a phase 2, double-blind, randomised, placebo-controlled trial. *Lancet.* (2017) 389:266–75. 10.1016/S0140-6736(16)32537-5 27988142

[31] ClinicalTrials.gov. *Filgotinib in the Induction and Maintenance of Remission in Adults With Moderately to Severely Active Crohn’s Disease (Diversity1).* (2020). Available online at: https://ClinicaltrialsGov/Ct2/Show/Nct02914561 (accessed December 9, 2022).

[32] ClinicalTrials.gov. *Filgotinib in Long-Term Extension Study of Adults with Crohn’s Disease (Diversitylte).* (2023). Available online at: https://ClinicaltrialsGov/Ct2/Show/Nct02914600 (accessed January 23, 2023).

[33] ClinicalTrials.gov. *Study to Evaluate the Efficacy and Safety of Filgotinib in the Treatment of Perianal Fistulizing Crohn’s Disease (Divergence2).* (2022). Available online at: https://ClinicaltrialsGov/Ct2/Show/Nct03077412 (accessed April 8, 2022).

[34] Danese S Vermeire S Zhou W Pangan A Siffledeen J Greenbloom S Upadacitinib as induction and maintenance therapy for moderately to severely active ulcerative colitis: results from three Phase 3, multicentre, double-blind, randomised trials. *Lancet.* (2022) 399:2113–28. 10.1016/S0140-6736(22)00581-5 35644166

[35] Vermeire S Colombel J Takeuchi K Gao X Panaccione R Danese S Dop38 upadacitinib therapy reduces ulcerative colitis symptoms as early as day 1. *J Crohn’s Colitis.* (2022) 16:i87–8. 10.1093/ecco-jcc/jjab232.077

[36] Long M Afzali A Fischer M Hudesman D Abdalla M McCabe R Tofacitinib response in ulcerative colitis (tour): early response after initiation of tofacitinib therapy in a real-world setting. *Inflamm Bowel Dis.* (2022). 10.1093/ibd/izac121 [Epub ahead of print]. 35700276 PMC10069660

[37] Burr N Gracie D Black C Ford A. Efficacy of biological therapies and small molecules in moderate to severe ulcerative colitis: systematic review and network meta-analysis. *Gut* (2021). 10.1136/gutjnl-2021-326390 [Epub ahead of print]. 34937767

[38] Lasa J Olivera P Danese S Peyrin-Biroulet L. Efficacy and safety of biologics and small molecule drugs for patients with moderate-to-severe ulcerative colitis: a systematic review and network meta-analysis. *Lancet Gastroenterol Hepatol.* (2022) 7:161–70. 10.1016/S2468-1253(21)00377-0 34856198

[39] Sandborn W Feagan B Loftus E Jr. Peyrin-Biroulet L Van Assche G D’Haens G Efficacy and safety of upadacitinib in a randomized trial of patients with Crohn’s disease. *Gastroenterology.* (2020) 158:2123–38.e8. 10.1053/j.gastro.2020.01.047 32044319

[40] ClinicalTrials.gov. *A Study of the Efficacy and Safety of Upadacitinib (Abt-494) in Participants with Moderately to Severely Active Crohn’s Disease Who Have Inadequately Responded to or Are Intolerant to Biologic Therapy.* (2022). Available online at: https://ClinicaltrialsGov/Ct2/Show/Nct03345836 (accessed August 15, 2022).

[41] ClinicalTrials.gov. *A Maintenance and Long-Term Extension Study of the Efficacy and Safety of Upadacitinib (Abt-494) in Participants with Crohn’s Disease Who Completed the Studies M14-431 or M14-433.* (2023). Available online at: https://ClinicaltrialsGov/Ct2/Show/Nct03345823 (accessed February 2, 2023).

[42] Barberio B Gracie D Black C Ford A. Efficacy of biological therapies and small molecules in induction and maintenance of remission in luminal Crohn’s disease: systematic review and network meta-analysis. *Gut.* (2023) 72:264–74. 10.1136/gutjnl-2022-328052 35907636

[43] Sands B Sandborn W Feagan B Lichtenstein G Zhang H Strauss R Peficitinib, an oral janus kinase inhibitor, in moderate-to-severe ulcerative colitis: results from a randomised, Phase 2 study. *J Crohns Colitis.* (2018) 12:1158–69. 10.1093/ecco-jcc/jjy085 29917064

[44] Sandborn W Nguyen D Beattie D Brassil P Krey W Woo J Development of gut-selective pan-janus kinase inhibitor Td-1473 for ulcerative colitis: a translational medicine programme. *J Crohns Colitis.* (2020) 14:1202–13. 10.1093/ecco-jcc/jjaa049 32161949 PMC7493219

[45] De Vries L Wildenberg M De Jonge W D’Haens G. The future of janus kinase inhibitors in inflammatory bowel disease. *J Crohns Colitis.* (2017) 11:885–93. 10.1093/ecco-jcc/jjx003 28158411 PMC5881740

[46] ClinicalTrials.gov. *Efficacy & Safety of Td-1473 in Ulcerative Colitis.* (2022). Available online at: https://ClinicaltrialsGov/Ct2/Show/Nct03758443 (accessed November 15, 2022).

[47] ClinicalTrials.gov. *Efficacy and Safety of Td-1473 in Crohn’s Disease (Dione).* (2022). Available online at: https://ClinicaltrialsGov/Ct2/Show/Nct03635112 (accessed January 27, 2022).

[48] Gu Y Sun W Zhang S Li X Wei W. Targeted blockade of Jak/Stat3 signaling inhibits proliferation, migration and collagen production as well as inducing the apoptosis of hepatic stellate cells. *Int J Mol Med.* (2016) 38:903–11. 10.3892/ijmm.2016.2692 27460897

[49] Chen B Zhong J Li X Pan F Ding Y Zhang Y Efficacy and safety of ivarmacitinib in patients with moderate-to-severe, active, ulcerative colitis: a phase ii study. *Gastroenterology.* (2022) 163:1555–68. 10.1053/j.gastro.2022.08.007 35963369

[50] ClinicalTrials.gov. *A Phase 3 Study to Investigate the Efficacy and Safety of Shr0302 with Moderately to Severely Active Ulcerative Colitis.* (2022). Available online at: https://clinicaltrialsgov/ct2/show/NCT05181137 (accessed September 9, 2022).

[51] ClinicalTrials.gov. *A Phase Ii Study in Patients with Moderate to Severe Active Crohn’s Disease.* (2022). Available online at: https://ClinicaltrialsGov/Ct2/Show/Nct03677648 (accessed June 6, 2022).

[52] ClinicalTrials.gov. *Study of Ost-122 in Patients with Moderate to Severe Ulcerative Colitis.* (2023). Available online at: https://ClinicaltrialsGov/Ct2/Show/ Nct04353791 (accessed January 13, 2023).

[53] Tokarski J Zupa-Fernandez A Tredup J Pike K Chang C Xie D Tyrosine Kinase 2-Mediated signal transduction in T lymphocytes is blocked by pharmacological stabilization of its pseudokinase domain. *J Biol Chem.* (2015) 290:11061–74. 10.1074/jbc.M114.619502 25762719 PMC4409266

[54] Papp K Gordon K Thaci D Morita A Gooderham M Foley P Phase 2 trial of selective tyrosine kinase 2 inhibition in psoriasis. *N Engl J Med.* (2018) 379:1313–21. 10.1056/NEJMoa1806382 30205746

[55] ClinicalTrials.gov. *An Investigational Study of Experimental Medication Bms-986165 in Participants with Moderate to Severe Crohn’s Disease.* (2023). Available online at: https://WwwClinicaltrialsGov/Ct2/Show/Nct03599622 (accessed January 12, 2023).

[56] ClinicalTrials.gov. *Safety and Efficacy of Deucravacitinib in Participants with Moderate to Severe Ulcerative Colitis.* (2023). Available online at: https://ClinicaltrialsGov/Ct2/Show/Nct03934216 (accessed February 21, 2023).

[57] Fensome A Ambler C Arnold E Banker M Brown M Chrencik J Dual inhibition of Tyk2 and Jak1 for the treatment of autoimmune diseases: discovery of ((S)-2,2-Difluorocyclopropyl)((1 R,5 S)-3-(2-((1-Methyl-1 H-Pyrazol-4-Yl)Amino)Pyrimidin-4-Yl)-3,8-Diazabicyclo[3.2.1]Octan-8-Yl)Methanone (Pf-06700841). *J Med Chem.* (2018) 61:8597–612. 10.1021/acs.jmedchem.8b00917 30113844

[58] Thorarensen A Dowty M Banker M Juba B Jussif J Lin T Design of a Janus Kinase 3 (Jak3) Specific Inhibitor 1-((2s,5r)-5-((7h-Pyrrolo[2,3-D]Pyrimidin-4-Yl)Amino)-2-Methylpiperidin-1-Yl)Prop-2-En-1-One (Pf-06651600) allowing for the interrogation of Jak3 signaling in humans. *J Med Chem.* (2017) 60:1971–93. 10.1021/acs.jmedchem.6b01694 28139931

[59] ClinicalTrials.gov. *Study to Evaluate the Efficacy and Safety of Oral Pf-06651600 and Pf-06700841 in Subjects with Moderate to Severe Crohn’s Disease.* (2022). Available online at: https://ClinicaltrialsGov/Ct2/Show/Nct03395184 (accessed September 23, 2022).

[60] ClinicalTrials.gov. *Study to Compare Oral Pf-06651600, Pf-06700841 and Placebo in Subjects with Moderate to Severe Ulcerative Colitis.* (2022). Available online at: https://ClinicaltrialsGov/Ct2/Show/Nct02958865 (accessed July 21, 2022).

[61] Winthrop K Melmed G Vermeire S Long M Chan G Pedersen R Herpes zoster infection in patients with ulcerative colitis receiving tofacitinib. *Inflamm Bowel Dis.* (2018) 24:2258–65. 10.1093/ibd/izy131 29850873 PMC6140434

[62] Colombel J. Herpes zoster in patients receiving jak inhibitors for ulcerative colitis: mechanism, epidemiology, management, and prevention. *Inflamm Bowel Dis.* (2018) 24:2173–82. 10.1093/ibd/izy150 29788127 PMC6140435

[63] Din S Selinger C Black C Ford A. Systematic review with network meta-analysis: risk of herpes zoster with biological therapies and small molecules in inflammatory bowel disease. *Aliment Pharmacol Ther.* (2022). 10.1111/apt.17379 [Epub ahead of print]. 36585944

[64] Al-Bawardy B Shivashankar R Proctor D. Novel and emerging therapies for inflammatory bowel disease. *Front Pharmacol.* (2021) 12:651415. 10.3389/fphar.2021.651415 33935763 PMC8080036

[65] Kim E Keam S. Filgotinib in rheumatoid arthritis: a profile of its use. *Clin Drug Investig.* (2021) 41:741–9. 10.1007/s40261-021-01055-0 34304373 PMC8613087

[66] Agrawal M Kim E Colombel J. Jak inhibitors safety in ulcerative colitis: practical implications. *J Crohns Colitis.* (2020) 14(Supplement. 2):S755–60. 10.1093/ecco-jcc/jjaa017 32006031 PMC7395307

[67] Spiewak T Patel A. User’s guide to jak inhibitors in inflammatory bowel disease. *Curr Res Pharmacol Drug Discov.* (2022) 3:100096. 10.1016/j.crphar.2022.100096 35300073 PMC8920857

[68] Lopez-Sanroman A Esplugues J Domenech E. Pharmacology and safety of tofacitinib in ulcerative colitis. *Gastroenterol Hepatol.* (2021) 44:39–48. 10.1016/j.gastrohep.2020.04.012 32829958

[69] Agouridis A Elisaf M Milionis H. An overview of lipid abnormalities in patients with inflammatory bowel disease. *Ann Gastroenterol.* (2011) 24:181–7. 24713706 PMC3959314

[70] Charles-Schoeman C Fleischmann R Davignon J Schwartz H Turner S Beysen C Potential mechanisms leading to the abnormal lipid profile in patients with rheumatoid arthritis versus healthy volunteers and reversal by tofacitinib. *Arthritis Rheumatol.* (2015) 67:616–25. 10.1002/art.38974 25470338 PMC5024065

[71] Chaparro M Acosta D Rodriguez C Mesonero F Vicuna M Acosta M Real-World evidence of tofacinitib in ulcerative colitis: short and long-term effectiveness and safety. *Am J Gastroenterol.* (2022). 10.14309/ajg.0000000000002145 36716287

[72] Olivera P Lasa J Bonovas S Danese S Peyrin-Biroulet L. Safety of janus kinase inhibitors in patients with inflammatory bowel diseases or other immune-mediated diseases: a systematic review and meta-analysis. *Gastroenterology.* (2020) 158:1554–73.e12. 31926171 10.1053/j.gastro.2020.01.001

[73] Charles-Schoeman C Wicker P Gonzalez-Gay M Boy M Zuckerman A Soma K Cardiovascular safety findings in patients with rheumatoid arthritis treated with tofacitinib, an oral janus kinase inhibitor. *Semin Arthritis Rheum.* (2016) 46:261–71. 10.1016/j.semarthrit.2016.05.014 27443588

[74] Sands B Taub P Armuzzi A Friedman G Moscariello M Lawendy N Tofacitinib treatment is associated with modest and reversible increases in serum lipids in patients with ulcerative colitis. *Clin Gastroenterol Hepatol.* (2020) 18:123–32.e3. 10.1016/j.cgh.2019.04.059 31077827

[75] Charles-Schoeman CG Choy E Camp H Song Y Anyanwu S McInnes I. Relationship between changes in lipid levels and improvement in disease activity outcomes in patients with rheumatoid arthritis receiving upadacitinib treatment: pooled analysis of data from two Phase 3 studies [Abstract]. *Arthritis Rheumatol.* (2020) 72.

[76] Husni ME Gladman D Helliwell P Van den Bosch F Tasset C Meuleners L Fri0344 the long-term effect of treating psoriatic arthritis with the Janus kinase 1-selective inhibitor filgotinib on lipid profiles: an analysis of the equator and equator2 trials. *Ann Rheumat Dis.* (2020) 79:766. 10.1136/annrheumdis-2020-eular.2494

[77] Sands B Colombel J Ha C Farnier M Armuzzi A Quirk D Lipid profiles in patients with ulcerative colitis receiving tofacitinib-implications for cardiovascular risk and patient management. *Inflamm Bowel Dis.* (2021) 27:797–808. 10.1093/ibd/izaa227 32870265 PMC8128390

[78] Charles-Schoeman C Fleischmann R Mysler E Greenwald M Wang C Chen A The risk of venous thromboembolic events in patients with ra aged ≥50 years with ≥1 cardiovascular risk factor: results from a phase 3b/4 randomized safety study of tofacitinib Vs Tnf inhibitors [Abstract]. *Arthritis Rheumatol.* (2021).

[79] Sandborn W Lawendy N Danese S Su C Loftus E Jr. Hart A Safety and efficacy of tofacitinib for treatment of ulcerative colitis: final analysis of octave open, an open-label, long-term extension study with up to 7.0 years of treatment. *Aliment Pharmacol Ther.* (2022) 55:464–78. 10.1111/apt.16712 34854095 PMC9300081

[80] Colombel JP Nakase H P573 the safety profile of upadacitinib maintenance therapy in ulcerative colitis in the phase 3 U-achieve study is consistent with that in approved indications. *J Crohns Colitis.* (2022) 16:i514–514. 10.1093/ecco-jcc/jjab232.699

[81] Mannucci A D’Amico F El Saadi A Peyrin-Biroulet L Danese S. Filgotinib for moderately to severely active ulcerative colitis. *Expert Rev Gastroenterol Hepatol.* (2022) 16:927–40. 10.1080/17474124.2022.2138857 36278878

[82] Wollenhaupt J Lee E Curtis J Silverfield J Terry K Soma K Safety and efficacy of tofacitinib for up to 9.5 years in the treatment of rheumatoid arthritis: final results of a global, open-label, long-term extension study. *Arthritis Res Ther.* (2019) 21:89. 10.1186/s13075-019-1866-2 30953540 PMC6451219

[83] Torres J Chaparro M Julsgaard M Katsanos K Zelinkova Z Agrawal M European crohn’s and colitis guidelines on sexuality, fertility, pregnancy, and lactation. *J Crohns Colitis.* (2023) 17:1–27. 10.1093/ecco-jcc/jjac115 36005814

[84] Selinger C Nelson-Piercy C Fraser A Hall V Limdi J Smith L IBD in pregnancy: recent advances, practical management. *Frontline Gastroenterol.* (2021) 12:214–24. 10.1136/flgastro-2019-101371 33912333 PMC8040511

[85] Mahadevan U Dubinsky M Su C Lawendy N Jones T Marren A Outcomes of pregnancies with maternal/paternal exposure in the tofacitinib safety databases for ulcerative colitis. *Inflamm Bowel Dis.* (2018) 24:2494–500. 10.1093/ibd/izy160 29982686 PMC6262193

[86] Tanaka Y Kavanaugh A Wicklund J McInnes I. Filgotinib, a novel Jak1-Preferential inhibitor for the treatment of rheumatoid arthritis: an overview from clinical trials. *Mod Rheumatol.* (2022) 32:1–11. 10.1080/14397595.2021.1902617 33740386

[87] Hellstrom W Dolhain R Ritter T Watkins T Arterburn S Dekkers G Manta and manta-ray: rationale and design of trials evaluating effects of filgotinib on semen parameters in patients with inflammatory diseases. *Adv Ther.* (2022) 39:3403–22. 10.1007/s12325-022-02168-4 35614292 PMC9239965

[88] Yates M Mootoo A Adas M Bechman K Rampes S Patel V Venous thromboembolism risk with jak inhibitors: a meta-analysis. *Arthritis Rheumatol.* (2021) 73:779–88. 10.1002/art.41580 33174384

[89] EMA. *Janus Kinase Inhibitors (JAKi).* (2023). Available online at: https://www.ema.europa.eu/en/medicines/human/referrals/janus-kinase-inhibitors-jaki (accessed February 22, 2023).

[90] Sandborn W D’Haens G Sands B Panaccione R Ng S Lawendy N Tofacitinib for the treatment of ulcerative colitis: an integrated summary of up to 7.8 years of safety data from the global clinical program. *J Crohns Colitis.* (2022). 10.1093/ecco-jcc/jjac141 [Epub ahead of print]. 36124702 PMC10069618

[91] Rubin D Reinisch W Greuter T Kotze P Pinheiro M Mundayat R Extraintestinal manifestations at baseline, and the effect of tofacitinib, in patients with moderate to severe ulcerative colitis. *Therap Adv Gastroenterol.* (2021) 14:17562848211005708. 10.1177/17562848211005708 34035832 PMC8132089

[92] Deodhar A Sliwinska-Stanczyk P Xu H Baraliakos X Gensler L Fleishaker D Tofacitinib for the treatment of ankylosing spondylitis: a phase III, randomised, double-blind, placebo-controlled study. *Ann Rheum Dis.* (2021) 80:1004–13. 10.1136/annrheumdis-2020-219601 33906853 PMC8292568

[93] Deodhar A Gensler L Sieper J Clark M Calderon C Wang Y Three multicenter, randomized, double-blind, placebo-controlled studies evaluating the efficacy and safety of ustekinumab in axial spondyloarthritis. *Arthritis Rheumatol.* (2019) 71:258–70. 10.1002/art.40728 30225992

[94] Paley M Karacal H Rao P Margolis T Miner J. Tofacitinib for refractory uveitis and scleritis. *Am J Ophthalmol Case Rep.* (2019) 13:53–5. 10.1016/j.ajoc.2018.12.001 30582071 PMC6288302

[95] Kochar B Herfarth N Mamie C Navarini A Scharl M Herfarth H. Tofacitinib for the treatment of pyoderma gangrenosum. *Clin Gastroenterol Hepatol.* (2019) 17:991–3. 10.1016/j.cgh.2018.10.047 30404036

[96] Chugh R Braga-Neto M Fredrick T Ramos G Terdiman J El-Nachef N Multicenter real-world experience of upadacitinib in the treatment of Crohn’s disease. *J Crohns Colitis.* (2022). 10.1093/ecco-jcc/jjac157 [Epub ahead of print]. 36272109

[97] Dalal R Mitri J Goodrick H Allegretti J. Real-World comparison of tofacitinib vs ustekinumab among bio-exposed patients with ulcerative colitis: a propensity score analysis. *Inflamm Bowel Dis.* (2021) 27:1694–7. 10.1093/ibd/izab097 33988235 PMC8522788

[98] Dalal RS Bains K One-Year comparative effectiveness of ustekinumab versus tofacitinib for ulcerative colitis after anti-tumor necrosis factor failure [Acg Abstract 42]. *Am J Gastroenterol.* (2022) 117(Suppl. 105). 10.14309/01.ajg.0000859512.98331.3f 36866198 PMC9972646

[99] Straatmijer T Biemans V Visschedijk M Hoentjen F de Vries A van Bodegraven A Superior effectiveness of tofacitinib compared to vedolizumab in anti-tnf-experienced ulcerative colitis patients: a nationwide dutch registry study. *Clin Gastroenterol Hepatol.* (2023) 21:182–91.e2. 35644343 10.1016/j.cgh.2022.04.038

[100] Amstad A Papagiannoulis E Scherer A Rubbert-Roth A Finckh A Mueller R Comparison of drug retention of tnf inhibitors, other biologics and jak inhibitors in ra patients who discontinued jak inhibitor therapy. *Rheumatology (Oxford).* (2022) 62:89–97. 10.1093/rheumatology/keac285 35579338

[101] Sandborn W Ghosh S Panes J Vranic I Wang W Niezychowski W A Phase 2 study of tofacitinib, an oral janus kinase inhibitor, in patients with Crohn’s disease. *Clin Gastroenterol Hepatol.* (2014) 12:1485–93.e2. 24480677 10.1016/j.cgh.2014.01.029

[102] Panes J D’Haens G Higgins P Mele L Moscariello M Chan G Long-Term safety and tolerability of oral tofacitinib in patients with Crohn’s disease: results from a phase 2, open-label, 48-week extension study. *Aliment Pharmacol Ther.* (2019) 49:265–76. 10.1111/apt.15072 30663107 PMC6646954

